# 1407. The Latent Tuberculosis Infection Cascade of Care during the COVID-19 Pandemic Response in a Mid-Sized US City

**DOI:** 10.1093/ofid/ofab466.1599

**Published:** 2021-12-04

**Authors:** Trevor M Stantliff, Lauren Houshel, Rinki Goswami, Serenity Millow, Gabrielle Cook, Robin Knapmeyer, Christa Easton, Jennifer Mooney, Moises A Huaman

**Affiliations:** 1 University of Cincinnati, Cincinnati, Ohio; 2 Hamilton County Public Health, Cincinnati, Ohio

## Abstract

**Background:**

The COVID-19 pandemic response may unintendedly disrupt multiple public health services, including tuberculosis control programs. We aimed to assess the cascade of care of latent tuberculosis infection (LTBI) in an urban US city during the COVID-19 pandemic response.

**Methods:**

We conducted a retrospective cohort study of adult patients who presented for LTBI evaluation at the Hamilton County Public Health Tuberculosis Clinic in Ohio between 2019 and 2020. We defined 01/2019 to 02/2020 as the pre-COVID-19 response period, and 04/2020 to 12/2020 as the COVID-19 pandemic response period. We reviewed electronic medical records and extracted sociodemographic information, medical history, and follow-up and treatment data to define steps within the LTBI cascade of care. Logistic regressions were used to assess factors associated with LTBI treatment acceptance and completion, adjusted by potential confounders and COVID-19 period.

**Results:**

Data from 312 patients were included. There was a significant decrease in the number of monthly LTBI referrals (median, 18 vs. 8, *p*=0.02) and LTBI evaluations (median, 17.5 vs. 7, *p*< 0.01) during COVID-19. There was a decrease in the proportion of immigrants as indication for LTBI testing (30% vs. 9%; *p*< 0.01), and an increase in LTBI diagnoses based on interferon-gamma release assay (IGRA; 30% vs. 49%; *p*< 0.01) during COVID-19. The proportion of people who were recommended LTBI treatment was similar before and during COVID-19 (76% vs. 81%, *p*=0.41), as well as the LTBI treatment acceptance rates (56% vs. 64%, *p*=0.28), and LTBI treatment completion rates (65% vs. 63%, *p*=0.85). In multivariate analysis, LTBI treatment acceptance was associated with Hispanic ethnicity, younger age, male sex, IGRA use, no comorbidities, and non-healthcare occupation, independent of COVID-19 period. LTBI treatment completion was associated with taking a rifamycin-containing regimen, independent of COVID-19 period.

**Conclusion:**

We observed a significant decline in the number of monthly LTBI referrals and evaluations during COVID-19. Our findings indicate an unintended negative impact of the COVID-19 response in LTBI screening efforts in our region. LTBI treatment acceptance and completion rates were not affected during COVID-19.

**Disclosures:**

**All Authors**: No reported disclosures

